# Farmworker Mobility and COVID-19 Vaccination Strategies: Yuma County, Arizona, 2021

**DOI:** 10.4269/ajtmh.22-0789

**Published:** 2024-04-23

**Authors:** Katherine A. Franc, Alba E. Phippard, Priscila Ruedas, Sarah J. Pinto, Kanan Mehta, Sonia Montiel, Sonia Contreras, Hannah Katz, Elvira McIntyre, Benito Lopez, Michelle Kreutzberg-Martinez, Daisy Steiner, Diana Gomez, Rebecca Merrill

**Affiliations:** ^1^ Division of Global Migration Health, Centers for Disease Control and Prevention, Atlanta, Georgia;; ^2^ Epidemic Intelligence Service, Centers for Disease Control and Prevention, Atlanta, Georgia;; ^3^ Yuma County Public Health Services District, Yuma, Arizona;; ^4^ Mel & Enid Zuckerman College of Public Health, University of Arizona, Tucson, Arizona;; ^5^ Kāpili Services, LLC, Orlando, Florida;; ^6^ Agency for Toxic Substances and Disease Registry, Geospatial Research, Analysis, and Services Program, Atlanta, Georgia;; ^7^ Arizona Department of Health Services, Phoenix, Arizona

## Abstract

Farmworkers, a group of essential workers, experience a disproportionately high burden of COVID-19 due to their living and working conditions. This project characterized farmworker mobility in and around Yuma County, Arizona, to identify opportunities to improve farmworker access to COVID-19 vaccination. We collected qualitative and geospatial data through a series of in-person and virtual focus group discussions, key informant interviews, and intercept interviews with participatory mapping. Participants included farmworkers, employers, and representatives of local institutions who serve or interact with farmworkers. We identified participants through purposive and referential sampling and grouped people by sociodemographic characteristics for interviews. We used qualitative and geospatial analyses to identify common themes and mobility patterns. The team interviewed 136 people from February 26 to April 2, 2021. Common themes emerged about how farmworkers have little or no access to COVID-19 vaccination unless offered at their workplaces or at locations where they congregate at convenient times. Further, farmworkers described how their demanding work schedules, long commute times, and caretaker commitments make it challenging to access vaccination services. Geospatial analyses identified three geographic areas in Yuma County where farmworkers reported living and working that did not have a COVID-19 vaccine clinic within walking distance. Coordination between local public health authorities and key partners, including employers and trusted representatives from local community-based organizations or the Mexican consulate, to offer vaccination at worksites or other locations where farmworkers congregate can help improve access to COVID-19 vaccines and booster doses for this population.

## INTRODUCTION

An estimated 2.8 million farmworkers are in the United States.^1^ These essential workers are disproportionately more affected by COVID-19 disease and associated hardships, often related to their substandard living and working conditions, such as inadequate access to healthcare and personal protective equipment and overcrowded workplaces and housing.^2^^,^^3^ In addition, farmworkers have an increased prevalence of underlying health conditions,^4^ including diabetes and other chronic illnesses, that put them at higher risk for severe outcomes and death from COVID-19.^3^

Farmworkers experience unique challenges in accessing COVID-19 vaccination owing to their mobility, cultural, and linguistic needs. Farmworkers who are H-2A visa holders^5^ are at increased risk for COVID-19 because of factors related to their living, working, and transportation conditions, lack of paid sick leave or medical insurance, underutilization of medical services, language and literacy barriers, and other factors.^6^ A national survey from 2017 to 2018 that focused on crop farmworkers in the United States who were non–H-2A visa holders found that 65% were born in Mexico, 18% were aged 55 years or older, 21% had family incomes below the poverty level, 64–65% preferred Spanish, 1–3% preferred an Indigenous language, and 13% were migrants (in this survey meaning that they traveled greater than 75 miles for a farm job).^7^ Migrant farmworkers who change geographic locations often for work have reduced healthcare utilization owing to difficulty knowing where health services are in unfamiliar locations, lack of transportation, and preferences to delay healthcare until returning to their permanent place of residence, such as Mexico.^8^ Farmworkers in the United States also have inadequate access to culturally and linguistically appropriate healthcare services, including vaccination.^9^

Yuma County, Arizona, along the United States–Mexico border and neighboring Imperial County, California, and Baja California and Sonora states in Mexico, has agriculture as its third largest employment sector.^10^ This large agricultural industry is supported by an estimated 38,000 migrant and seasonal farmworkers and 46,008 nonworking dependents per year.^11^ However, this most recent population estimate for migrant and seasonal farmworkers, which is from 2014, is outdated, and much of the knowledge about the Yuma County farmworker population that exists among local community-based organizations and other partners is not formally documented. A representative of Yuma County Public Health Services District (YCPHSD) explained that when COVID-19 vaccines first became available, vaccine allocations for Yuma County were based on census data that did not account for mobile populations who are part-time residents, such as migrant and seasonal farmworkers, winter visitors, or U.S. citizens who reside in Mexico and cross the border daily for work in agriculture, healthcare, long-term care facilities, education, childcare, and other professions. This led to an allocation that was smaller than the population eligible to receive COVID-19 vaccines and to delays in moving through phases of COVID-19 vaccine prioritization^12^ for higher risk populations (D. Gomez, E-mail communication, December 11, 2023). Collection and consolidation of information about mobile populations can aid public health officials in planning responses for COVID-19 and other public health emergencies, tailored to a local community’s true population size and specific needs.

Considering the above, the YCPHSD, Arizona Department of Health Services (AZDHS), and Centers for Disease Control and Prevention (CDC) partnered to characterize farmworker mobility in and around Yuma County and identify opportunities to improve farmworker access to COVID-19 vaccination. This collaborative team (hereafter referred to as the “team”) aimed to understand farmworker mobility patterns, community congregation points, farmworker access to COVID-19–related health services and information, and perceptions about COVID-19 vaccination among farmworkers, their employers, and organizations that support farmworkers. The primary objective of this assessment was to aid public health authorities in and around Yuma County in planning and delivering COVID-19 public health interventions, especially vaccinations, in their jurisdiction.

## MATERIALS AND METHODS

### Qualitative data collection.

The project team, which included local health department staff with experience working with the Yuma County farmworker community and its partners, adapted the Population Connectivity Across Borders (PopCAB)^13^^,^^14^ toolkit to collect data on farmworker mobility in and around Yuma County, Arizona, and farmworkers’ COVID-19 vaccine perceptions, enablers, and barriers. The PopCAB toolkit,^13^^,^^14^ developed by the CDC, includes qualitative and geospatial methods such as focus group discussion (FGD) guides, key informant interviews (KIIs), surveys and participatory mapping used to evaluate population mobility, and community access to public health services among mobile populations to enable public health partners to integrate information about population mobility into programming and resource allocation decisions. The FGD guides were not piloted with local farmworkers before data collection began because we used an open-ended style of interviewing. However, the team adapted the FGD guides using feedback from local health department staff with personal experience working with the Yuma County farmworker community and its partners, who were also family members of farmworkers. Interviewers engaged in a dialogue with participants, refining questions in real time and asking follow-up questions to elicit information.

The team implemented FGDs, KIIs, and intercept interviews (IIs) with farmworkers and local partners knowledgeable about mobility patterns and access to public health interventions (e.g., testing, vaccination, healthcare) for the farmworker community. Focus group discussions involved two to eight participants in group interviews lasting 60–90 minutes. Key informant interviews were interviews with a single informant lasting 30–60 minutes. Intercept interviews were brief, 5–15-minute interviews with a single participant or group interviews with two to four interviewees using a set of priority questions. Intercept interview length depended on participant availability and surrounding circumstances, such as length of work breaks. All interviews, including the FGDs, were completed between February 26 and April 2, 2021.

Trained team members facilitated virtual or in-person discussions using modified PopCAB tools that included questions adapted for each participant group (Supplemental Materials 1–3). Topics included farmworker mobility patterns; specific locations of community congregation points; barriers, opportunities, and trusted sources for accessing COVID-19 testing, vaccination, and health information; and perceptions about COVID-19 vaccination. For the purposes of this project, “farmworker” refers to people working in many different types of agricultural jobs for cultivating, harvesting, processing, and packaging crops and animal products. Facilitators led the discussions in the participants’ preferred language, Spanish or English. Discussions were audio recorded and transcribed by notetakers in the language spoken. Throughout implementation, the team adapted the question guides based on participant responses and interviewed participants until saturation was achieved and no new themes emerged.^15^

Participant recruitment. The team collaborated with local partners to identify the types of participants and community-based organizations or other partners to invite to participate. The YCPHSD, Mexican consulate, employers, and farmworker-serving organizations helped recruit participants by mixed methods, including purposive and referential/snowball sampling. Partners recruited participants who self-identified as farmworkers having worked at any time in Yuma County or who were knowledgeable about farmworker health or mobility patterns in and around Yuma County (i.e., employers, labor contractors, farmworker family members, community health workers [CHWs]). The team did not offer interpretative or translation services to non-Spanish and non-English speakers, and participants were recruited in Spanish or English. Thus, people who spoke only Mesoamerican Indigenous or other languages were not included. Partners identified participants as H-2A visa holders during recruitment. Immigration status was not collected during interviews unless voluntarily disclosed. Participants were adults, 18 years of age or older. Farmworkers, farmworker family members, and CHWs were offered a $25 gift card for their participation.

### Situational considerations.

Participants were grouped by sociodemographic characteristics (i.e., farmworker or relative of farmworker, employer or supervisor, diplomatic, community-based organization, academic, or immigration authority) for interviews when possible. The team aimed to implement interviews this way to promote candid information sharing among peers and to mitigate the influence of potential power dynamics and distrust between people of different groups, such as those that exist between employers and employees. When possible, a trusted community messenger, such as a representative from the local Mexican consulate, was present during the interviews with farmworkers to help build trust between facilitators and participants.

The team conducted virtual interviews when possible (i.e., participant[s] had easy access to a computer and internet) to avoid risks of COVID-19 transmission. When virtual interviewing was not possible or if participants preferred in-person interviews or did not have access to a computer and internet for virtual interviews, the team implemented COVID-19 health and safety protocols to prevent transmission among participants and facilitators. These measures included temperature checks; verbal screening of COVID-19 signs, symptoms, and exposure; required face covers; physical distancing; regular disinfection of hands and shared surfaces; and use of well-ventilated outdoor spaces.

### Qualitative data analysis.

The team used qualitative analyses to identify themes, subthemes, and mobility patterns that emerged from the FGD, KII, and II data. Because transcripts were recorded in the language that was spoken, fluent Spanish speakers analyzed Spanish transcripts and English speakers analyzed English transcripts. Both deductive and inductive analysis methods were applied to identify themes and subthemes by eight team members who analyzed the transcripts. Discrepancies between reviewers were resolved through group discussion and consensus. In addition, when applicable, one local team member who is bilingual, bicultural, and native to the local community interpreted the community-specific context and meaning of phrases and expressions.

### Geospatial methods and analysis.

Interviews also included participatory mapping^14^^,^^16^ to gather geospatial data about mobility patterns and community congregation points from every participant group and from intercept interviewees. The team created a database in a Microsoft Excel spreadsheet of all geospatial data related to points of interest, congregation points, and mobility patterns gathered from maps annotated by participants and interview transcripts. The team digitized, mapped, and analyzed the spatial data using Esri’s ArcGIS software (Esri, Redlands, CA). The team used the spatial data to complete a service area network analysis to better understand the accessibility of vaccine providers from farmworker residences and housing locations identified during interviews. Using Esri’s StreetMap Premium 2020 road network, the team calculated areas within 15- and 30-minute walking times from farmworker residences or housing locations. Publicly accessible data on vaccine provider locations sourced from ArcGIS Online were overlaid on maps along with walking time polygons. The team used Esri’s ArcGIS Desktop 10.8 for all mapping and analysis.^17^

### Ethics.

This activity was reviewed and approved as nonresearch by the CDC, AZDHS, and YCPHSD. Participation was voluntary and anonymous with no names or identifiable information recorded about participants. All participants provided verbal consent to participate, and sessions were audio recorded when participants gave permission.

## RESULTS

The team implemented 15 FGDs, 6 KIIs, and 31 IIs with 132 participants from February 26 to April 2, 2021 (Table 1). Participants included farmworkers or their family members, employers or agricultural industry representatives, CHWs, and leadership from local institutions, such as farmworker-serving organizations, public health services, consular services, immigration authority, local university academics, and others (Table 1).

**Table 1:** Profile of population connectivity across borders participants by interview type for activities completed in Yuma County, February 26–April 2, 2021

Partner Group*	Focus Group Discussion	Key Informant Interview	Intercept Interview^†^	Total Number of Participants
Farmworkers or their family members	6 (49)^‡^	0 (0)	29 (44)	93
Community health workers (promotores/promotoras de la salud)	2 (11)	0 (0)	0 (0)	11
Agricultural employers or industry representatives (including labor contractors)	4 (9)	1 (1)	0 (0)	10
Yuma county local health department (Yuma county public health services district)	1 (6)	0 (0)	0 (0)	6
Leadership of local farmworker-serving organizations	0 (0)	2 (2)	0 (0)	2
Academics in local, higher education institutions (public health or agriculture departments)	1 (2)	2 (2)	0 (0)	4
Local port (immigration) authority	0 (0)	1 (1)	0 (0)	1
Local consulate of Mexico	1 (3)	0 (0)	0 (0)	3
Manager of local business frequented by farmworkers	0 (0)	0 (0)	1 (1)	1
Local elected official	0 (0)	0 (0)	1 (1)	1
Total	15 (80)	6 (6)	31 (46)	132

* Interviews with farmworkers, farmworker family members, and community health workers were conducted in person. Interviews with other partner groups were a mix of in-person and virtual formats.

^†^ Intercept interviews are defined as brief 5–15-minute interviews with one to four interviewees using a set of priority questions.

^‡^ No. of events (No. of total participants).

Seventy percent of participants (70%, *N* = 93/132) were Yuma County farmworkers or their family members. Among farmworker participants, 59% were male, 18% were H-2A visa holders, and 45% had a fieldwork type of job (cutting, harvesting, packing, loading, or irrigation) (Table 2).

**Table 2:** Demographic characteristics of farmworker participants—farmworker mobility and COVID-19 vaccination strategies—Yuma County, Arizona, 2021 (*N* = 93)

Characteristic	Frequency (*n*)	Percentage* (%)
Sex		
Female	38	41
Male	55	59
Age (years)		
18–29	23	25
30–59	54	58
60+	16	17
Job type		
Fieldwork (cutting, harvesting, packing, loading, irrigation)	42	45
Machine operator	12	13
Truck driver	3	3
Quality control	3	3
Supervisor	3	3
Family member	1	1
Other^†^	6	6
Unknown (blank or illegible)	23	25
H-2A visa holder^‡^		
Yes	17	18
Unknown (blank)	76	82

* Percentages for each category may not add up to 100% because values are rounded to nearest whole number.

^†^ Other responses for job type included general laborer, equipment maintenance, cashier, cleaning, and clerk.

^‡^ Participants were identified as H-2A visa holders by partners during recruitment; immigration status was not confirmed during interviews unless voluntarily disclosed.

The top three overarching themes that emerged from the qualitative analysis were the following: Farmworkers are a highly mobile population, farmworkers experience many barriers to accessing COVID-19 vaccination, and employers and other local partners play a major role in enabling access to COVID-19 vaccination for this community. These themes were evident across all types of participant groups and are described further in the following sections. Unless a particular participant group is specified, the theme was common among all types of participant groups.

### Farmworker mobility.

**Figure 1.**
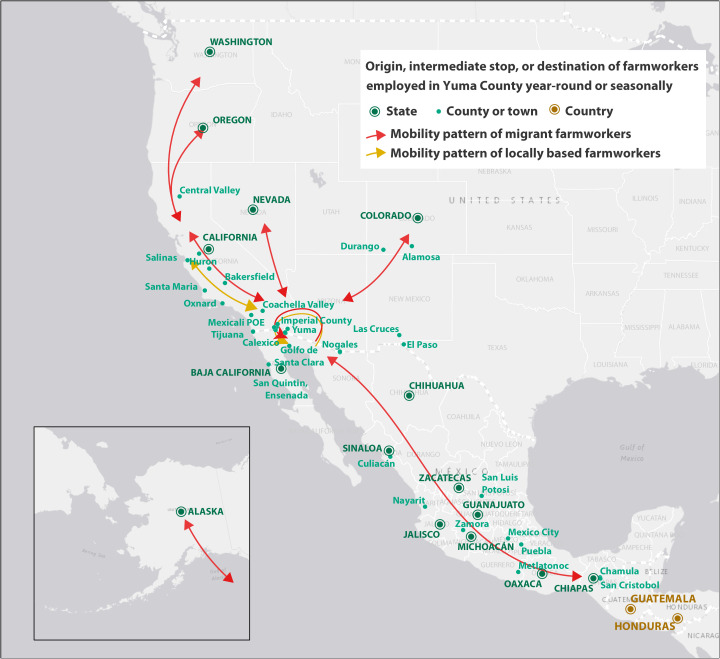
Movement patterns of Yuma County farmworkers, Yuma, Arizona, based on community engagement activities in Yuma County in 2021. This figure shows location data gathered during community engagement activities in Yuma County in 2021. Farmworkers who work in Yuma County at least sometime during the year are a highly mobile population. Participants described and annotated on maps the complex movement patterns and trends of farmworkers, indicated by arrows, as well as their origins, destinations, and intermediate stops, indicated by yellow and green dots. Yellow arrows indicate movement patterns of local farmworkers who worked and lived in Yuma County or nearby jurisdictions on either side of the U.S.–Mexico border, usually within a 50-mile radius. Red arrows indicate movement patterns of migrant farmworkers who temporarily relocated to fill surge workforce needs during peak harvest and growing seasons. Red and yellow circular arrows indicate pendulous, or frequent, movement back and forth across the U.S.–Mexico border that is common for all types of farmworkers, including local and migrant workers and workers who cross the U.S.–Mexico border regularly, sometimes daily, to get to and from home and work.

Farmworkers who work in Yuma County at least sometime during the year are a highly mobile population. Participants described and annotated on maps the complex movement patterns and trends of farmworkers, as well as their origins, destinations, and intermediate stops (Figure 1). Participants described many different types of farmworker mobility patterns, which included local, migrant, and pendulous types of movement. Local movement patterns include those farmworkers who worked and lived in Yuma County or nearby jurisdictions, on either side of the U.S.–Mexico border (e.g., municipality of San Luis Río Colorado in Mexico), usually within a 50-mile radius (Figure 1, yellow arrows). Migrant movement patterns include workers who temporarily relocated to “follow the crops,” or “siguen la corrida,” such as H-2A visa holders, to fill surge workforce needs during peak harvest and growing seasons (Figure 1, red arrows). Pendulous movement patterns include workers who crossed the U.S.–Mexico border regularly, and sometimes daily, to get to and from home and work (Figure 1, red and yellow circular arrows), such as the daily border–crossing population, or for other travel reasons. The daily border-crossing population was estimated by participants to be about 15,000 farmworkers per day. These farmworkers include workers with an H-2A visa, lawful permanent residents (i.e., “green card” holders), U.S. citizens who live in Mexico, and other people crossing the border.

Farmworker movement patterns are not mutually exclusive of each other. Both local and migrant workers in Yuma County move pendulously across the U.S.**–**Mexico border for many reasons, such as to visit friends and family and seek health services or traditional medicine in Mexico that is more affordable, accessible, or culturally familiar, especially for those who are uninsured. Participants from farmworker-serving organizations reflected that the border-crossing population of farmworkers does not usually include unauthorized workers, who actively avoid the scrutiny of government agencies, particularly border authorities.

Workforce needs fluctuate throughout the year, bringing workers into and out of the local area to live and work at different times depending on the industry. In Yuma County, the largest demand for local and migrant labor is during the winter leafy greens season, which begins around late September and continues through April. Participants reflected on how farmworker mobility may vary during a season as a result of changes in the weather, delays in the visa process, and changing market demands for crops. Aside from the farmworkers themselves, precise details about farmworker mobility, such as the timing and size of the workforce that is arriving and departing during different times of the year, are best known by employers, who are often agriculture labor contractors.

**Figure 2.**
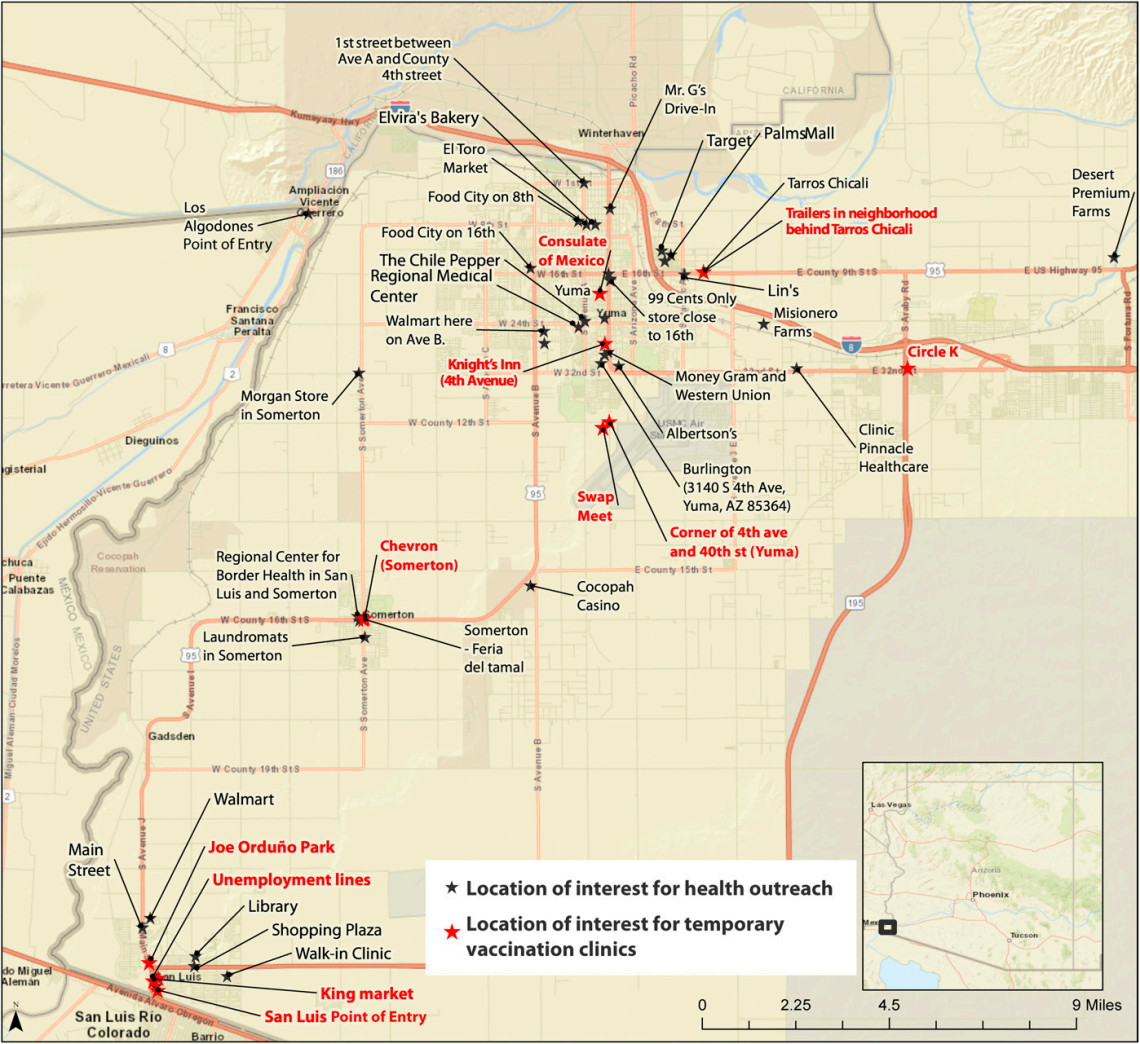
Locations of interest for temporary or mobile vaccination sites and/or health outreach in downtown Yuma and San Luis based on community engagement activities in Yuma County in 2021. This figure shows location data gathered during community engagement activities in Yuma County in 2021. The black and red stars indicate a location frequented by farmworkers. The red stars indicate locations most suitable for temporary or mobile vaccination clinics because of the high volume of farmworkers at these locations at predictable times. Many of these sites may also be ideal for other forms of health outreach, such as risk communication or health education.

Participants identified numerous locations that are favorable for public health outreach and mobile vaccination clinics because they are frequented by large numbers of farmworkers at predictable times. These include residential areas popular among farmworkers (both employer- and nonemployer provided), transportation hubs, the local port of entry (i.e., border crossing), and destinations for shopping, recreation, leisure, and healthcare (Figure 2).

### Farmworker barriers to accessing COVID-19 interventions.

Participants described many barriers that farmworkers experience when accessing COVID-19 interventions, including vaccinations. These include barriers that all types of farmworkers experience, such as demanding work schedules and home commitments, long commute times, and discrimination and job insecurity. These barriers make it challenging, and sometimes impossible, for farmworkers to access vaccination services outside their workplaces. Some farmworkers, such as H-2A visa holders, experience additional barriers such as living and working in rural locations and being dependent on employers for housing and transportation, which limits their access to health and other social services without support from their employers.

Participants described how farmworkers’ demanding work schedules, long commute times, and caretaker commitments make it challenging for them to access vaccination and other health services. Many farmworkers spend several hours waiting in daily border lines to get to and from work and have work schedules that include working in the middle of the night to avoid the heat; starting their workday at 1 a.m., typically 6 and sometimes even 7 days a week; and working long hours such as 12+-hour workdays. Many farmworkers also serve as caregivers for dependent children and elderly or other family members. Their schedules do not allow them to go to vaccine appointments during regular business hours, when most clinics and pharmacies offer these appointments, and they do not have the financial security to miss hours of pay. A farmworker’s typical workday in Yuma County includes waking up at 1 a.m., waiting 1**–**4 hours in pedestrian or vehicle lines to cross the U.S.–Mexico border into the United States, working 12 or more hours, crossing the U.S.–Mexico border again to return to Mexico, sleeping for a few hours at home, and repeating the cycle.

“Some [farmworkers] only spend 4 hours at home per day, the rest of their time is spent commuting or working in the fields.” – **Manager of a local business popular among farmworkers**

“I cross the border every day for work [on a motorcycle, which is faster than waiting in the pedestrian line]… Get up at 3 a.m., in line around 4 a.m., wait for 1 and a half to 2 [hours]…” [translated from Spanish]. – **Local farmworker**

“Farmworkers do not have vehicles, so this is a barrier – have to bring resources to the workers. Sometimes they do not have time, they earn income for the amount of work that they do, if they leave their place of work to do something else then they lose money. It is not that they are not health conscious, but it is a more urgent priority to get money and they cannot leave work.” – **Mexican consular representative**

Job insecurity and discrimination that farmworkers experience reduce uptake of COVID-19 vaccinations and other public health services. During the FGDs with local community-based and academic institutions serving farmworkers, participants shared how farmworkers feel “replaceable,” and some believe that accessing public services (e.g., COVID-19 vaccinations), taking sick leave, going to the doctor, or disclosing symptoms of COVID-19 may lead their employer to perceive them as an “inconvenience” and may compromise their job security. Although their legal status was not discussed in FGDs with farmworkers, some participants shared that some workers, including unauthorized workers, are known to avoid seeking public services, including health services such as COVID-19 vaccination, and limit their movement within communities for fear of coming to the attention of immigration authorities and being deported.

“Farmworkers are ‘replaceable,’ and they know that. If they can’t do the job, then the employer will find someone who can do it. If they are sick or feel sick or cough, then farmworkers do whatever they can so people do not know that they are sick.” – **Representative of a local farmworker-serving organization**

“Many [farmworkers] do not want to take the test [for COVID-19] because they lose a day of work and if you lose a day of work, you lose your place. And if you come out positive, you can’t go [to work for] 2 weeks they don’t know if they’re going to pay you. And if you rest for 2 weeks, you may lose your job… I think there is a great lack of resources to bring the tests to a time and a place that is convenient for the field workers because if the tests start at 7 to 8 [a.m.], they are already working.” [translated from Spanish] – **Community health worker**

Community health workers (also known in Spanish as promotores de la salud), the Mexican consulate, and faith-based leaders are trusted sources of information among Yuma County farmworkers. Community health workers provide outreach to farmworkers, such as mobile vaccine services or assistance with H-2A hiring, at their worksites and other locations at times and places convenient for the workers. Farmworker employers act as “gatekeepers” to health, social, and community services that are provided by trusted messengers because permission is needed to access workplace and housing premises. Many farmworkers live in employer-provided housing in remote locations that are hard to reach, work long and changing work hours, or may have curfews imposed by their employers, all of which make it challenging for trusted messengers to reach farmworkers without employer permission. This is especially true for H-2A workers who depend on their employers for housing and transportation or those farmworkers who live and work in rural locations of the county. Compared with non–H-2A farmworkers, H-2A workers were reported as experiencing even greater physical, social, and cultural isolation from their peers, CHWs, and other trusted community messengers.

“En realidad es el sistema que ellos tienen así como más aislados, como protegidos; incluso hay áreas donde allí les dan de comer para que no salgan… donde por ejemplo el patrón que los contrata tiene áreas donde ellos viven allí, duermen allí, se les cocinan allí.” This translates to “In reality it’s the system that has them [the H-2A workers], like, more isolated, like protected [from the outside]; there are even places where they [the employer] feed them so they don’t go out…where for example the employer that hires them has areas where they live there, they sleep there, they cook for them there.” – **Community health worker**

**Figure 3.**
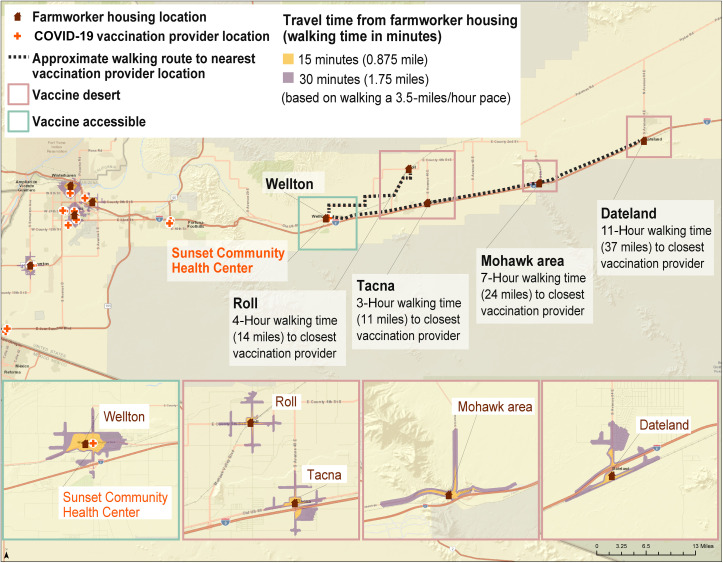
Vaccine deserts: Walking time from farmworker housing location to COVID-19 vaccine clinics in rural communities, Yuma County, Arizona, based on community engagement activities in Yuma County in 2021. Geospatial analyses of walking times to COVID-19 vaccine points of distribution in the community identified three geographic areas in Yuma County that can be considered “vaccine deserts,” or places where farmworkers reported to live and work that did not have a vaccine point of distribution within walking distance (<30 minutes), which is a barrier, as many farmworkers have limited transportation to travel farther than walking distance from their residence or workplace.

Geospatial analyses of walking times to COVID-19 vaccine points of distribution in the community identified three geographic areas in Yuma County that can be considered “vaccine deserts,” or places where farmworkers have been reported to live and work that did not have a vaccine point of distribution within walking distance (Figure 3). Vaccine deserts are a barrier because many farmworkers have limited transportation to travel more than walking distance from their residence or workplace.

### Farmworker enablers for accessing COVID-19 vaccination.

Participants indicated that farmworkers’ employers are a major source of health information and access to public health services for farmworkers. Workplaces were reported to use a variety of platforms to disseminate health information to their employees, including in-person meetings, workforce-wide text messages, employer-sponsored mobile phone applications, posters and signs in the workplace, and the use of shift managers or work crew (“cuadrilla”) supervisors to reinforce health messages during work hours. Participants said this helps reach employees without a phone or computer.

When asked about where they get trusted sources of health information, a farmworker participant stated: “from my employer, they provide an orientation every year when we arrive, it includes information about pesticide safety and other topics. This year it included COVID [translated from Spanish],” whereas another participant stated, “lo que sale del trabajo, la compañía nos manda por texto o a veces llamadas,” which translates to “what comes from my employer, the company sends by text messages or sometimes phone calls.” – **Local farmworker(s)**

Interviewed employers were motivated to support their employees in getting vaccinated for COVID-19. However, some employers reflected that they or their peers did not know they could solicit the health department or other local partners to help coordinate vaccination clinics or other COVID-19 interventions for their workforce.

“The agriculture industry has a huge interest in getting their workers vaccinated… They have offered to have bus driven to spot where they can be vaccinated as a crew… They know vaccine appointments will not work… There is no way for a farmworker to get there.” – **Academic in a local institution of higher education**

For those workers who have limited proficiency in English or Spanish, their work shift leaders (“mayordomos”) or a colleague can usually serve as translators and communicate important messages to them. Nearly all farmworkers in Yuma County speak Spanish. However, the level of Spanish comprehension may be limited for some. There are few workers who speak Indigenous languages, such as Quiche, Maya, Tzotzil, Mixteco, and Mixteco Alto.

Participants shared that most farmworkers would be accepting of and willing to get the COVID-19 vaccine if their employers offered it at the workplace or coordinated transportation for them to receive it during work hours. Farmworkers were motivated to get vaccinated because of the effect COVID-19 has had on their families and friends, as well as to protect others around them. In fact, some said that seeing the negative impact of COVID-19 on their families or communities motivated them to get vaccinated.

“Employees [farmworkers] that have access and eligibility to getting vaccinated on their own, sooner, would rather wait for the employer to provide the opportunity for them to get vaccinated. This may be preferred because of ease of access, ability to be vaccinated “on the clock” – they [the farmworkers] are busy and do not want to deal with the process of getting vaccinated, and if they get side effects the employer will know the cause.” – **Local agricultural industry representative**

When asked about the choice to get vaccinated, one farmworker participant stated, “Protegerse a uno, proteger a las demás personas que estamos aquí todos juntos en el trabajo, para el bien de todos,” which translates to “Protect oneself, protect the others that are all here together at work, for the good of everyone.” – **Local farmworker**

Participants from employer and farmworker FGDs who had already coordinated or participated in workplace vaccination events noted that social and peer pressure in the workplace to get vaccinated improved acceptance for some who were initially hesitant. Worksite vaccination clinics generated a dialogue about the vaccine among peers and, for some, built vaccine confidence over time.

*A farmworker participant shared their experience that*, “Antes de vacunarme estaba entre sí y entre no [indeciso]. Ví que mis compañeros lo hicieron y me animé también,” which translates to “Before getting vaccinated I was between yes and no [undecided]. I saw that my colleagues did it [got vaccinated], and I was encouraged, too.” – **Local farmworker**

Participants shared how specific responses to misinformation through in-person discussion with trusted community messengers can improve vaccine acceptance among farmworkers who are initially hesitant. Some farmworkers or farmworker family members shared their own concerns about the COVID-19 vaccine or those they had heard about from colleagues, friends, family members, or online (Table 3). They expressed the importance of providing opportunities for farmworkers and their family members to voice their concerns and ask questions about the vaccine in person with trusted health messengers.

**Table 3:** Concerns among the farmworker community about the COVID-19 vaccine

COVID-19 Vaccine Concern, Hesitancy, or Misinformation Summarized into an Overarching Theme	Selected Quote(s) from Interviews as Spoken (Spanish)	English Translation of Quote
Waiting to consult with their medical providers or religious leaders before vaccination	“Yo comenté que no quiero ponérmela [la vacuna] por alergia a la penicilina… Voy a hablar con la clínica y preguntar.” “Yo voy a la iglesia cristiana y el pastor nos da la información de síntomas.”	“I commented that I don’t want to get it [the vaccine] because of my allergy to penicillin… I’m going to talk to the clinic and ask.” “I go to the Christian church, and the pastor gives us symptom information.”
Fear, suspicion, mistrust, and misunderstanding about the vaccine development process (e.g., concerns about how rapidly the vaccines were developed)	“La vacuna es algo nuevo; fue un modelo nuevo que aventaron así; yo, meter algo al cuerpo, si no estoy 100% seguro, no lo hago; y cuando yo me pongo nervioso…me llegaron ansiedades…me dije na, no me lo pongo.”	“The vaccine is something new; it was a new model that they threw out like that; for me, putting something in the body, if I’m not 100% sure, I don’t do it; and when I get nervous…I get anxieties… I told myself nah, I won’t take it.”
Fear, suspicion, misunderstanding, and ethical concerns about the vaccine itself (e.g., that it could cause changes to a person’s physical features or DNA or the belief that vaccine ingredients include aborted fetal cells or implants [i.e., microchips] to monitor the population)	“Una tía me dijo que es un plan del gobierno que ya nos quiere ir eliminando y luego allá en Estados Unidos dicen que van a tener a la gente vigilada.” “A mí, lo me motiva es porque he escuchado que las vacunas tienen células de fetos abortados. Yo no estoy de acuerdo en el aborto. Es opuesto a mis creencias.”	“One aunt told me that it is a government plan that wants to eliminate us, and later, there in the United States, they say that they are going to have the people monitored.” “I have heard that vaccines have cells from aborted fetuses. I do not agree with abortion. It’s opposed to my beliefs.”
Vaccine side effects and concerns about subsequently missing work	“Yo no me quiero vacunar porque todas las personas que la ha recibido [la vacuna] están saliendo enfermos.”	“I don’t want to get vaccinated because all the people who have received it [the vaccine] are becoming sick.”
Beliefs that vaccine efforts are part of a government plan to reduce fertility in the population	“En la política se habla, que esto pasa cada 100 años para disminuir la población, para controlar la economía.”	“In politics, it is said that this happens every 100 years to reduce the population, to control the economy.”
Religious beliefs that getting the vaccine is a way of marking people and that people should not allow it	“Incluso una compañera estaba nerviosa porque una sobrina, que está muy apegada a la iglesia, le mandó un mensaje diciendo “tía, no te vacunes porque eso en la biblia está que es como una marca que le van a poner a la gente.”	“A friend was nervous because a niece, who is very close to the church, sent her a message saying, ‘Aunt, don’t get vaccinated because in the Bible it’s said that it’s like a mark that they are going to put on people.’”

Data were collected through interviews with farmworkers and their family members or local partners serving farmworkers in Yuma County, Arizona, from February 26 to April 2, 2021.

### Other sources of health information.

Social media and internet searches (Google) were common, though not widely trusted, sources of information for farmworkers and their family members. Participants described Facebook groups specifically for the local farmworker community or for daily border crossers working in agriculture and widely circulated WhatsApp posts as an important method of sharing information within these communities. These media sources are useful mechanisms for disseminating COVID-19–related information in the Yuma County farmworker community. Farmworkers also mentioned local TV networks and Spanish news and radio as a source of information for COVID-19 updates.

## DISCUSSION

Through qualitative and spatial data collection with farmworkers, employers, farmworker-serving organizations, and other partners in Yuma County, Arizona, in early 2021, this project identified important barriers and opportunities to improve COVID-19 vaccination access and coverage among the farmworker community. For this mobile population with limited ability to travel during work or nonwork hours to receive a vaccine, vaccine availability at vaccination sites in local healthcare facilities, pharmacies, and other points of dispensing was not directly associated with vaccine accessibility for farmworkers. This may lead to low COVID-19 primary and booster vaccination coverage among farmworkers and could contribute to the disproportionately high burden of COVID-19 illness and death among this population.

Our findings indicate that most farmworkers have little or no access to COVID-19 vaccination unless offered at their workplaces or at a convenient time and location(s) for them because they work long hours, and many have limited transportation to travel more than walking distance from their residence or workplace. Further, most farmworkers prefer to get vaccinated with assistance from their employer, through workplace vaccination events or employer-coordinated transportation during work hours to vaccine points of distribution, and would not be able get vaccinated otherwise because of the previously mentioned barriers.

Local health departments, employers, community-based organizations, Mexican consulates, and other partners can coordinate to offer vaccination and other public health interventions at worksites or other convenient locations where farmworkers congregate to help improve access for this population during public health emergencies. These public-private partnerships to improve vaccine accessibility for farmworkers are important for several reasons. First, by expanding collaboration with private industry, such as employers and agriculture labor contractors, health departments can reach more farmworkers to improve accessibility to the COVID-19 vaccine and booster doses. Employers know when their workforce, including new workers, are arriving and departing from the local area, so workplace vaccination events can be scheduled to reach the most farmworkers at the right times. For workers who may not be reachable through their employers, such as when employers are uninterested or if their contact information is unknown, vaccination events can be offered at locations and times where farmworkers congregate outside of work to improve accessibility (Figure 2). Local health departments may be able to build a network of employers and labor contractors by collaborating with local industry associations, which include farms and organizations as members by type of commodity (e.g., local growers association). Second, this project highlighted that employers are a major source of access to health information and COVID-19 interventions. Health departments and farmworker-serving organizations can work directly with employers to ensure that the information provided is up-to-date and linguistically and culturally accessible for all farmworkers. Third, public health officials can facilitate connecting trained and trusted health messengers with farmworkers before or during worksite vaccination events. If a trusted source (i.e., a health messenger) can be trained and available on-site to answer questions before or during a worksite vaccination event, this can help to improve vaccine acceptance even further. Fourth, power imbalances between farmworkers and employers due to job insecurity and discrimination that farmworkers are subjected to makes it crucial for local public health authorities and other trusted local partners (i.e., CHWs, consular services) to work directly with employers to establish a workplace culture where all farmworkers are informed of their rights and have support and confidence to access timely and quality health and other public services. The enablers and barriers to accessing public health services that we identified for farmworkers are similar to those reported in previously published literature.^2^^–^^4^^,^^6^^,^^8^^,^^9^ Coordinated efforts to build trust and inform workers of their rights to access public health services irrespective of their immigration, occupational, or residency status is an important part of ensuring coverage and equity for these communities on the move.

### Strengths and limitations.

There are many strengths of this project. First, there was rich community engagement and leadership from local partners to design the project and recruit participants. Second, bilingual and bicultural facilitators interviewed participants in their preferred language, Spanish or English. Third, diverse partner groups who interact with, use, or serve farmworkers were interviewed to triangulate findings and solicit many different perspectives. Fourth, when possible, a trusted community representative was trained to facilitate or introduce facilitators to farmworkers to help build trust and promote candid information sharing. Fifth, we gathered spatial data through participatory mapping to better characterize mobility and geographic factors related to vaccine accessibility. This included creating maps to reflect information about congregation points and community points of interest to support the health department for informational and planning purposes.

The limitations of this work include having supervisors or employer representatives present for some IIs and FGDs with farmworkers, which may have limited honest responses to interview prompts and possibly introduced bias. More specifically, when interviews with farmworkers took place at worksites or employer-provided housing accommodations, it was sometimes unclear if supervisors or a company health and safety representative was present part of or the entire time as a spectator, and this was not systematically notated in transcripts when it occurred. This prevented us from stratifying results by this variable during analysis (supervisor present: yes/no) to identify and adjust for potential response bias. COVID-19 health and safety protocols (physical distancing, mask wearing, and videoconferencing when possible) created challenges and barriers between facilitators and may have reduced participant engagement. Likewise, language barriers may have affected the participation of Indigenous farmworkers with limited Spanish proficiency as participant recruitment, FGDs, and IIs were conducted in Spanish, and translation services for non-Spanish or non-English speakers were unavailable. Finally, because we used referential or snowball sampling,^18^ selection bias in employers is possible. Employers were recruited through previous contact with the health department or referrals from other interviewees, and our employer participants may have been more likely to have positive attitudes toward workplace vaccination events. However, other participant groups, such as farmworkers, were recruited by a mix of referrals from CHWs, employers, and the Mexican consulate in Yuma, so they were unlikely to be biased in favor of or against workplace vaccination.

## CONCLUSION

This work aimed to compile and consolidate local knowledge from the farmworker community and the partners who employ or serve them about how to provide equitable access to prevention measures during public health emergencies, such as vaccination during the COVID-19 pandemic. The project focused on a local transborder community, like many others with an agriculture economy, with changing population size and complex farmworker mobility patterns that fluctuate depending on seasonal farm labor needs. It is important to note that mobile populations, including farmworkers, are not often included in population estimates used to allocate public health resources, such as annual census data. As a result, jurisdictions may not receive adequate funding, vaccines, or other resource allocations to provide public health interventions to these mobile populations. Understanding and characterizing mobility trends of farmworkers, such as in this project, and estimating the size and mobility of permanent and nonpermanent populations during different times of the year can help with public health preparedness planning, especially interstate and international coordination of mass vaccination campaigns or other public health services during public health emergencies. In summary, coordination between local health departments, employers, community-based organizations, the Mexican consulate, and other local partners to meet farmworkers when and where they are geographically, to offer trusted and culturally appropriate health outreach and vaccination services at worksites or other convenient locations, can contribute to increased coverage and uptake for COVID-19 vaccines and booster doses among farmworkers.

“A donde andan, vamos,” which translates to “Where they go, we go.” – **Community health worker**

## Supplemental Materials

10.4269/ajtmh.22-0789 Supplemental Materials
