# Supplementary material for: Farmworker Mobility and COVID-19 Vaccination Strategies: Yuma County, Arizona, 2021
Source: Am J Trop Med Hyg. 2024 Apr 23;110(6):1180–90. doi: 10.4269/ajtmh.22-0789 (PMC11154059; doi:10.4269/ajtmh.22-0789)
Supplement: Supplemental Materials [file tpmd220789.SD1.pdf]

S1. Questions and discussion topics for non-farmworker participants.

**Theme or Topic of Interest**	**Questions for all partner groups participating in KIIs or FGDs***<br>**Underlined: priority questions; lettered a-d: optional question probes*
**Role**	1. Please describe your role within your agency or organization; OR, your background as it relates to engaging with farmworkers in and around **[PLACE]**.<br>• For example, for farmworker employers; what types of commodities are farmed, how are farmworkers hired, and where are farms located? For farmworker serving organizations, what types of services are provided, for how many farmworkers, and how are services delivered?
**Agency priorities and priority geographic areas**	2. Please describe your agency's/organization's/establishment's priorities related to **COVID-19** and farmworkers.<br>3. In which geographic areas or among which farmworker groups are these priorities relevant? Why?<br>• Example reasons for why a geographic area or farmworker group may be considered high priority:<br>- COVID-19 testing or vaccine hesitancy<br>- Limited access to healthcare resources (geographic isolation, cost, etc.)<br>- Large healthcare facility that attracts farmworkers from across the region<br>- Limited access to support from COVID-19 relief funds (e.g. due to visa or residency status)

	- Marginalized groups of farmworkers with low visibility in COVID-19 surveillance system<br>- Highly mobile groups of farmworkers<br>- High-density of farms or other establishments hiring farmworkers<br>- Residential areas commonly occupied by farmworkers and their dependents<br>- Establishment/business that has frequent contact with particular group, or in particular areas
**Trends and patterns of farmworker movement in and beyond [PLACE].**	4. Using the map, please identify and describe areas of high priority when you consider where farmworkers move to, from, and through **[PLACE]**.<br>a. Why are these areas high priority?<br>• Example reasons for why an area may be considered high priority:<br>- High-density of farms or other establishments hiring farmworkers<br>- Residential areas commonly occupied by farmworkers and their dependents<br>- Common transportation routes or methods used by farmworkers<br>5. Using the map, please describe **local, interstate, intrastate**, and **international** farmworker movement patterns. Discuss briefly **who,**

	**why, to/from, when, how and how many** farmworkers are moving in, around and beyond **[PLACE]**.<br>a. Where do the migrant farmworkers go after they complete their work? Other parts of **[PLACE]**, **[PLACE]**, the US, or Mexico?<br>b. Describe the characteristics of farmworkers who travel into/through/out of this area? (c*haracteristics include country of origin, occupation or specific skills, cultural identity, language, etc.*)<br>c. Do different nationalities, or cultural and linguistic groups go to different places?<br>d. Do new farmworkers come often or do the same farmworkers routinely travel into/through/out of these areas and return to the area?
**Occupation/industries**	6. What work/activities do farmworkers come to do in **[PLACE]**?<br>a. Planting, harvesting, packaging, multiple, other types?<br>b. Which crops and farms do they work with?<br>c. Which part of the county do they work in?
**Seasonality**	7. Describe the daily, weekly, seasonal movement patterns for farmworkers in **[PLACE]**.<br>a. When (what time of the year) do they come to **[PLACE]**? Why?<br>b. What times of year sees the most farmworkers?<br>c. Are farmworkers in the area year-round?<br>d. How long do farmworkers stay in the area? Why?

	e. Does this differ in different parts of the county? Are there different parts of the county that receive more farmworkers in certain months or seasons?<br>f. Do the same groups (nationalities, or ethnic/cultural/linguistic groups) come throughout the year or do different groups tend to come different times of the year?<br>g. Do the same individuals in these groups tend to come year after year or are there different groups that come each year?<br>• For example, can we expect the workers that are here this March to be here next March?
**Factors influencing farmworker movement**	8. <u>What factors are driving farmworker movement (i.e. economic, environmental, socio-cultural, conflict, others)?</u><br>a. What are reasons and frequency of farmworker movement across the US-Mexico border?<br>b. What are reasons and frequency of farmworker movement between neighboring states and jurisdictions around **[PLACE]**?<br>c. Describe why farmworkers visit or stay in **[PLACE]**.<br>i. Reasons may include:<br>- Seeking healthcare (for specific illnesses or treatment?)<br>- Buying and selling of goods (what types of goods?)<br>- Religious or cultural practices (festivals, holidays, or routine practice?)<br>- Professional or economic reasons (markets, livelihood opportunities)

	- Education (boarding schools, primary schools, etc.)<br>- Long-distance migration<br>- Family connections (regular visits, special occasions, or funerals)<br>- Others?<br>ii. Additional probing questions:<br>- Are the services more accessible to farmworkers in a particular geographic area? If so, which ones (social, healthcare, housing, etc.)? What makes them more accessible (hours/location/other)?<br>- Is travel to this area easier than to another area with similar services?<br>- Where appropriate, do rules and regulations result in more or less travel to this place?<br>- How have patterns of farmworker mobility changed with the COVID-19 pandemic?<br>- What are the average wait times at the US-Mexico border? Has this changed with COVID-19? If so, how?<br>d. Are there any trends in farmworker mobility by type of visa or immigration/residency status? If so, what are they?<br>i. Are there any data sources exist that describe this?<br>e. What factors (environmental or otherwise) might influence the number of migrant farmworkers that come to **[PLACE]** or the timing of when they arrive or depart?

	9. <u>Do farmworkers tend to live and work in the same locations over the various season?</u><br>a. Where are those locations?<br>b. When, why and where do farmworkers live in **[PLACE]** during various seasons?<br>c. When, why and where do farmworkers live in **Mexico** during various seasons?<br>10. <u>Are there any special places, festivals, events that attract migrant farmworkers throughout the year in</u> **[PLACE]** <u>or nearby?</u><br>a. What are the main congregation points for farmworkers? Why?
**Linguistic and cultural needs**	11. <u>Where do the farmworkers come from and what languages do they speak?</u> Describe in as much detail as possible the areas of origin (state, municipio, ciudad, colonia) and specific population demographics.<br>12. <u>How do farmworkers consume information and where do they seek out updates?</u> (COVID-19 related, or work related?) ie: WhatsApp groups (faith-based, family chats), Facebook groups, radio, tv, posters, etc.?<br>a. For farmworkers that speak indigenous languages, are you aware of communications or messaging targeting those populations in their native languages? If yes, what topics does the messaging address, and how is the content generated?
**Modes and routes of travel**	13. <u>How and where do farmworkers move within and beyond</u> **[PLACE]**<u>?</u>

	b. For what reasons, how many, and how often do farmworkers living in and around **[PLACE]** use the **[PLACE]** Port of Entry?<br>c. Why might farmworkers regularly or occasionally cross the border to/from the area? (seeking health care, livelihood, etc.)<br>d. Describe what means of transportation farmworkers take to reach the location, e.g. train, rideshare, bicycle, by foot, etc.<br>e. What are the common transit points in **[PLACE]** used by farmworkers?<br>f. Do farmworkers use different crossings to leave the country than they did to enter it? If yes, why? E.g. do they use different crossings to enter the country and leave or vice versa.
**Additional priority areas**	After discussing all priority areas, ask the following question.<br>14. <u>Please identify additional geographic areas you feel are of public health interest based on farmworker movement patterns. Describe why these areas are important.</u>

S2. Partner-specific discussion topics and questions for non-farmworker participants.

<table>
<tr><th>Participating Partner</th><th>Additional questions to ask during KII or FGDs</th></tr>
<tr><td>Health Department</td><td>1. What is your current strategy for vaccinating farmworkers?
a. What challenges do you anticipate?
2. Where do farmworkers go to seek healthcare services or information about diseases? Why do they seek services from these avenues?
3. How does the Health Department communicate with mobile farmworkers?</td></tr>
<tr><td>Farmworker Employers</td><td>1. How has COVID-19 in your workforce impacted your business?
a. Have you changed your hiring/transportation/housing practices since COVID-19 started?
2. What coordination exists between you and public health authorities to implement COVID-19 control measures to protect the health of your workforce? How can this be strengthened?
3. Can you describe the immigration or residency status distribution of farmworkers you hire? For other employers in [PLACE]?
a. For example: how many are H2A visa holders versus legal permanent residents (green card holders) versus US citizens versus others?</td></tr>
<tr><td>Community Health Workers</td><td>1. What health services do you provide to farmworkers? How and where do you reach them?
2. What are the 3 biggest challenges you anticipate to vaccinating farmworkers in and around [PLACE]? What support do you need for this?</td></tr>
</table>

	3. What do farmworkers think about COVID-19 testing and vaccination?<br>4. Where do farmworkers go to seek healthcare services or information about diseases? Why do they seek services from these avenues?<br>5. Are there any sub-groups of the farmworker population that CHW's have a difficult time reaching? Or geographic areas that are difficult to provide services to? Why?<br>6. What health conditions are most common among farmworkers?<br>7. What health services do farmworkers have access to?
**Local Farmworker Serving Organization Leadership**	1. What coordination exists between you and public health authorities to implement COVID-19 control measures to protect farmworker health? How can this be strengthened?<br>2. Where do farmworkers go to seek healthcare services or information about diseases? Why do they seek services from these avenues?<br>3. How does your organization communicate with mobile farmworkers?<br>a. Can you describe the immigration or residency status distribution of farmworkers in **[PLACE]** For example: how many are H2A visa holders versus legal permanent residents (green card holders) versus US citizens versus others?<br>4. What types of health beliefs or attitudes exist among farmworkers regarding to COVID-19 testing and vaccination?
**Port Authority**	1. What types of changes in farmworker movement might you anticipate seeing over the next 1-2 months? Year? Both within **[PLACE]**, and in or out of **[PLACE]**? Why?

	2. Have you observed any changes in farmworker movement across the border during the pandemic? With the new administration change?<br>3. How, if at all, does CBP change its activities or otherwise prepare for anticipated changes in farmworker movement or respond to unanticipated changes?<br>4. *For an informant that regularly observes the border crossings, and assuming we can't get this info from other CBP sources:* Can you estimate the number of farmworkers that cross the border from MX daily during this current growing season? Throughout the year?<br>5. Where are CBP border checkpoints located in and around **[PLACE]**?<br>a. How do you think these checkpoints influence farmworker movement? Please describe for formal and informal checkpoints.
**Mexican Consulate**	1. Where do farmworkers go to seek healthcare services or information about diseases (specify agency, and county if needed)?<br>a. Why do they seek services from these avenues?<br>b. How might this differ for the different ethnic, cultural, or linguistic groups of farmworkers?<br>2. Are there seasonal or demographic patterns in farmworkers seeking services at Mexican consulates and Ventanillas de Salud (VdS)? What about the consulate's Mobile Health Units (MHU's)?<br>3. Can you estimate the # of farmworkers the consulate/Ventanillas/MHU's serve daily during different times of the year?<br>a. What are the reasons farmworkers seek these services?

	b. Does this vary depending on the time of year? If so, how?<br>c. Are there particular groups of farmworkers that seek services through these avenues?<br>4. Are farmworkers referred to the VdS for services by any other groups or organizations? Do VdS refer farmworkers to particular clinics or other providers? If so, where? How many?<br>5. Can you describe coordination between Mexican and US- public health authorities or NGO's (in **[PLACE]**) related to the health of mobile farmworkers and COVID-19? Coordination could serve to provide farmworkers with a place to isolate or quarantine, PPE, testing, or vaccination, or COVID-19 health information/education, for example.<br>a. Do you have specific mechanisms for coordination for mitigating COVID-19 in farmworkers? If so, what are they?<br>b. How many farmworkers are served through these mechanisms for coordination? Where do these activities take place?<br>c. Do you think this coordination could be improved? If so, how?<br>d. Do any challenges or barriers exist? If yes, what are they?<br>6. What are farmworker perceptions about vaccination? About COVID-19?<br>7. What types of services do farmworkers seek from these avenues?
**Farmworker Serving Organizations**	1. What services do you provide for farmworkers?<br>2. How do the farmworkers and farmworker families your program serves move throughout the year?<br>a. When are they arriving, leaving, staying; how many?<br>b. Where are they going?

	3. How many families with these types of movement patterns do you serve?<br>4. Do any of the farmworkers bring dependents, family members, or other companions with them when they migrate? Do these family members move with farmworkers and follow their seasonal 'corridor' of work or do they stay put in **[PLACE]** for longer periods of time?<br>5. How are services or locations designed to accommodate mobility of farmworkers and their families?<br>a. How do children get to the **[PLACE]** centers daily? By what modes of transportation?<br>b. Do you have any awareness, or can you estimate how many children (under 18) perform farm work in **[PLACE]**?<br>6. Can you describe the immigration or residency status distribution of farmworkers in **[PLACE]** County?<br>a. For example: how many are H2A visa holders versus legal permanent residents (green card holders) versus US citizens versus others?<br>7. How many farmworker families does your organization currently serve? How might you expect that number (i.e. need for services) to change in the next several months? Why?<br>8. Have the **[PLACE]** Centers remained open during various COVID-19 'stay at home' orders? Does your organization provide any health-related outreach or services for families?

**Academia – Agriculture**	1. Can you describe how farms and the farming workforce is distributed in and around **[PLACE] ?**<br>2. How are farmworkers hired by establishments in **[PLACE]**? Is there any seasonal variation (where possible, please be specific in terms of numbers of farmworkers, commodity being farmed, and location)?<br>3. What types of changes in farmworker movement might you anticipate seeing over the next 1-2 months? Year? Both within **[PLACE]**, and in or out of **[PLACE]**? Why?<br>4. Have you observed any changes in farmworker movement across the border during the pandemic? With the new administration change?
**Academia – Public Health**	1. How do you expect trends in farmworker movement in and around **[PLACE]** to change in the coming months? Next year? Next 2-5 years or long-term? Why?<br>2. What types of health beliefs or attitudes exist among farmworkers regarding to COVID-19 vaccination?<br>3. Are there trends in farmworker mobility by type of visa or immigration/residency status?<br>a. What data sources exist that describe this?
Farmworker-serving businesses: **Grocery stores**	1. How do farmworkers get to and from your store to buy food?
Farmworker-serving businesses:	1. What modes of transportation are used by farmworkers? Where are they going? When and how often?

**Transportation services**	
Farmworker-serving businesses: **Housing (motels, other residences)**	1. What types of living accommodations are used by farmworkers?<br>a. How many farmworkers use each type of living accommodation? Does this vary by season? If so, how?<br>b. Do farmworkers use more than one type of living accommodation? If so, when and what types? Please describe.<br>2. How do farmworkers travel from their living accommodations to work?
Farmworker-serving businesses: **Market vendors**	1. Are there any services or vendors related to health, that farmworkers can find at this market?<br>a. If so, what types of services? Who provides these services? Where do they provide these services?<br>b. Are there vendors that sell traditional health, folk or home remedies at the market? If so, what might they be? Do they also sell their products during the week at other locations? If so, where are those shops?
Farmworker-serving businesses: **Financial institutions**	1. What are the major reasons farmworkers use your businesses' services? Does this vary during different times of the year?<br>a. Are there particular groups of farmworkers that use your services, for example, those who traveled to **[PLACE]** from outside the border region?

**Farmworker serving faith-based organizations**	1. Do you provide health information to your congregants or community?<br>a. If so, what does this consist of?

S3. Questions and discussion topics for farmworker participants.

Theme or Topic of Interest	Questions for all partner groups participating in KIIs or FGDs*<br>**Underlined: priority questions; lettered a-d: optional probes*
**Participant introductions**	1. <u>Please describe who you are and your background. Where are you from? What brings you here if you are not from</u> <u>**[PLACE]**?</u><br>• Each participant, as interested, should describe their background as it relates to the design of the focus group discussion. For example, what types of farms do they work on? What are their jobs (picking produce, sorting, packing, tending to animals, etc.)? Encourage participants to be specific and describe all jobs held throughout the year.<br>• Use this question to begin orienting participants to the map.
**Trends and patterns of participant movement in and beyond [PLACE] County.**	2. <u>Please identify and describe, using the map, areas of interest you visit in this general area (</u>**[PLACE]** <u>and surrounding jurisdictions; show map)?</u><br>• Purpose: Gather introductory information on participants' geographical sense of community and the areas they often visit, including where they live. Inform participants that the locations do not need to be confined to short distance travel or areas shown on the map. They should include locations they travel to near and far for any reason, including other states and Mexico.<br>a. Where do you work? How long have you worked there (or each place if multiple)?

	b. How long have you been a farmworker?<br>c. Where do you travel? Why do you travel? Do you travel for any other reasons such as personal or for family?<br>d. Where are you from or where were you born? How often do you visit your home village/town and why?<br>e. Where does your family reside? How often do you visit them or they visit you?<br>f. Where do you often shop, eat and stay overnight?<br>g. Where do you go to the doctor? If you have gotten tested for COVID-19, where did you get tested?<br>h. Do you seek other care such as folk or home remedies or traditional medicine?<br>i. Other frequent activities? Such as religious or cultural activities? Shopping? Others?<br>3. Which of these identified areas of interest do you feel are also important for other farmworkers? Why are these areas important?<br>• Purpose: Gather information on the level of importance of the identified areas of interest for farmworkers in **[PLACE]**. Aim to document on the map all the major identified areas and transit points along travel routes.<br>4. Please describe the **local, interstate, intrastate**, and **international** movement of yourself and your farmworker peers. Discuss briefly **who, why, to/from, when, how and how many** of your farmworker peers are moving in, around and beyond **[PLACE]**.

	5. Where do you and your peers go after completing your work? Other parts of **[PLACE]**, **[PLACE]**, the US, or Mexico?<br>a. Describe the characteristics of your peers who travel into/through/out of this area? (c*haracteristics include country of origin, occupation or specific skills, cultural identity, language, etc.*)<br>b. Do new farmworkers come often or do the same farmworkers routinely travel into/through/out of these areas and return to the area?
**Occupation/industries**	6. What work/activities do you and your peers who are farmworkers come to do in **[PLACE]**?<br>a. Planting, harvesting, packaging, multiple, other types?<br>b. Which crops and farms do you or your peers work with?<br>c. Which part of the county do you or your peers work in?
**Seasonality**	7. Please describe your daily, weekly, seasonal patterns for visiting or traveling through this area.<br>a. When (what time of the year) do you or your peers come to **[PLACE]**? Why?<br>b. What times of year are you or your peers in **[PLACE]**?<br>c. Are you or your peers in the area year-round?<br>d. How long do you or your peers stay in the area? Why?<br>e. Does this differ in different parts of the county? Are there different parts of the county that have more farmworkers in certain months or seasons?

	f. Do the same groups (nationalities, or ethnic/cultural/linguistic groups) come throughout the year or do different groups tend to come different times of the year?<br>g. Do the same individuals in these groups tend to come year after year or are there different groups that come each year?<br>• For example, can we expect the workers that are here this March to be here next March?
**Factors influencing movement**	8. Why do you and your farmworker peers visit or stay in **[PLACE]**?<br>a. What are reasons and frequency you move across the US-Mexico border and between neighboring states and jurisdictions to and from **[PLACE]** County?<br>iii. Reasons may include:<br>- Going to the doctor (for specific illnesses or treatment?)<br>- Buying and selling of goods (what types of goods?)<br>- Religious practices (festivals, holidays, or routine practice?)<br>- Work or economic reasons (markets, livelihood opportunities)<br>- Education (boarding schools, primary schools, etc.)<br>- Family connections (regular visits, special occasions, or funerals)<br>- Others?<br>iv. Additional probing questions:

	- Are the services more accessible to you or your peers in a particular geographic area? If so, which ones (food, immigration services, healthcare, housing, etc.)?<br>- Why are they more accessible? Is travel to this area easier than to another area with similar services? Is it more accessible with your work schedule (location/hours)?<br>- Where appropriate, do rules and regulations result in more or less travel to this place?<br>- How have your patterns of visiting or traveling through this area changed with the COVID-19 pandemic?<br>b. Are there any trends in farmworker mobility by type of visa or immigration/residency status? If so, what are they?<br>c. What factors (environmental or otherwise) influence if you come to **[PLACE]** and the timing of when you arrive or depart?<br>9. <u>Do you or your peers tend to live and work in the same locations over the various season?</u><br>a. Where are those locations?<br>b. When, why and where do you or your peers live in **[PLACE]** during various seasons?<br>c. When, why and where do you or your peers live in **Mexico** during various seasons?<br>10. <u>Are there any special places, festivals, events that you or your peers attend throughout the year in</u> **[PLACE]** <u>or nearby?</u>

	a. How long do you or your peers stay in the area before of after the event?<br>b. Where do you gather with others in the area? Why?
**Linguistic and cultural needs**	11. <u>Where do your farmworker peers come from and what languages do they speak?</u><br>12. <u>How do you receive information and where do you seek out updates?</u> (COVID-19 related, or work related?) ie: WhatsApp groups (faith-based, family chats), Facebook groups, radio, tv, posters, etc.?<br>a. If you or your peers speak indigenous languages, are you aware of communications or messaging in your native languages? If yes, what topics does the messaging address, and where does the content come from?
**Modes and routes of travel**	13. <u>How and where do you and your peers who work on farms move into, within and beyond **[PLACE]**?</u><br>a. For what reasons, and how often do you and your peers use the **[PLACE]** Port of Entry?<br>b. Why do you or your peers regularly or occasionally cross the border to/from the area? (seeking health care, livelihood, etc.)<br>c. How do you travel in and beyond **[PLACE]**? Under what circumstances do you use different forms of transportation? E.g. train, rideshare, bicycle, by foot, etc.<br>d. What are the common transit points in **[PLACE]** used by you or your peers?

	e. Do you or your peers use different crossings to leave the country than you do to enter it? If yes, why? E.g. do they use different crossings to enter the country and leave or vice versa.
**Additional priority areas**	After discussing all priority areas, ask the following question.<br>14. <u>Please identify additional areas you feel are important to you or your peers in **[PLACE]**.</u>
**Additional questions**	15. Where do you get COVID-19 health information?<br>16. What do you think or feel about COVID-19 testing?<br>a. Is COVID-19 testing accessible to you? If so, where and when?<br>17. What do you think or feel about COVID-19 vaccination?<br>a. Is the vaccine available to you? If so, where and when? If not, do you know when it will be available to you?<br>b. Do you plan to get the vaccine?<br>c. Where and how would it be easiest for you to get the vaccine?<br>18. About how many of your peers who are farmworkers in and around **[PLACE]** are H2A visa holders versus legal permanent residents (green card holders) versus US citizens versus have other immigration or residency status?<br>19. What are the top 1-3 challenges or hardships you are experiencing now with COVID-19?<br>a. For example: Finding safe housing, work, or transportation? Obtaining protective equipment like face covers? Accessing COVID-19 testing, or others? Please describe.
